# Comparative accuracy of photon-counting and dual energy CT pulmonary angiography perfusion mapping using V/Q scans as reference standard

**DOI:** 10.1007/s10140-026-02479-x

**Published:** 2026-04-25

**Authors:** Alexis Ogbonna, Jordan H. Chamberlin, Dhiraj Baruah, Cesar Lopez Rodriguez, Ian Mencken, Jeremy R. Burt, Saeed Elojeimy, Ismail M. Kabakus

**Affiliations:** 1 Division of Cardiothoracic Imaging, Department of Radiology, Medical University of South Carolina, 96 Jonathan Lucas Street Suite 210, MSC 323, Charleston, SC 29425 USA; 2 Division of Cardiothoracic Radiology, Department of Radiology and Imaging Sciences, University of Utah, Salt Lake City, UT USA; 3 Division of Nuclear Medicine, Department of Radiology, Medical University of South Carolina, Charleston, SC USA

**Keywords:** Photon-counting detector CT, Dual-energy CT, Lung perfusion, V/Q SPECT/CT, Spectral imaging

## Abstract

**Purpose:**

To compare the diagnostic performance of photon-counting–detector CT (PCD-CT) and dual-energy CT (DECT) perfusion maps against ventilation–perfusion (V/Q) SPECT/CT as reference standard across diverse clinical indications, and to provide quantitative iodine density reference values.

**Methods:**

This retrospective study included two independent cohorts who underwent V/Q SPECT/CT and either PCD-CT or DECT. Lobar perfusion was assessed on each modality by two blinded readers with consensus serving as the reference for inter-reader agreement. Diagnostic accuracy, sensitivity, specificity, PPV, and NPV were calculated using V/Q SPECT/CT as the reference standard. Iodine density was measured using standardized regions of interest placed in normally perfused and hypoperfused lobes. Inter-reader agreement was evaluated using Cohen’s κ. Statistical comparisons were performed using paired or unpaired tests as appropriate, with significance set at *p* < 0.05.

**Results:**

PCD-CT demonstrated an accuracy of 0.85, sensitivity of 0.71, specificity of 0.97, and κ of 0.80. DECT demonstrated an accuracy of 0.89, sensitivity of 0.52, specificity of 0.97, and κ of 0.59. Differences in diagnostic performance between PCD-CT and DECT were not statistically significant. Iodine density was significantly higher in normally perfused lung compared with perfusion defects for both PCD-CT (2.10±0.29 vs 0.40±0.18 mg/mL; *p* < 0.001) and DECT (2.30±0.29 vs 0.49±0.17 mg/mL; *p* < 0.001).

**Conclusion:**

Both PCD-CT and DECT provide high diagnostic accuracy for detecting lobar perfusion defects, with PCD-CT demonstrating higher inter-reader agreement. This study provides the first quantitative iodine reference values for PCD-CT pulmonary perfusion, supporting its potential as a robust functional complement to conventional CTPA.

## Introduction

The functional assessment of pulmonary perfusion plays a critical role in the evaluation of multiple cardiopulmonary diseases including pulmonary embolism (PE), chronic thromboembolic pulmonary hypertension (CTEPH), sickle cell disease (SCD), and small vessel vasculopathies. Ventilation–perfusion (V/Q) scintigraphy, particularly when combined with single-photon emission computed tomography (SPECT), is widely recognized for its high sensitivity and specificity in diagnosing perfusion abnormalities, particularly in the context of PE and CTEPH [1]. V/Q scans are also frequently used in the workup of the diseases listed above, as well as assessing functional perfusion for pre-transplant surgical planning. However, V/Q imaging has notable limitations, including relatively low spatial resolution, long acquisition times, dependence on radiopharmaceutical availability, and patient compliance constraints [2]. During their workup, many of these patients also undergo computed tomography pulmonary angiography (CTPA) exams to aid in their overall diagnosis.

Dual-energy CT (DECT) has emerged as an alternative CT-based approach to generate pulmonary perfusion maps derived from iodine distribution. By acquiring data at two energy spectra, DECT can estimate iodine concentration, providing pulmonary perfusion map that has been proven to demonstrate strong correlation to V/Q scans in the detection of perfusion defects [3]. This has been increasingly adapted in clinical practice for the diagnostic work-up of acute and chronic PE, sickle cell disease, pulmonary hypertension, interstitial lung diseases, and lung function pre- and post-procedure [4].

In addition to dual-energy CT, spectral imaging can also be achieved using detector-based techniques, such as dual-layer (sandwich) detector CT systems. In these systems, a single x-ray source is used, and spectral separation is achieved at the detector level by stacking two scintillation layers that differentiate high- and low-energy photons. This approach enables retrospective spectral analysis from a single acquisition, allowing generation of iodine maps and perfusion imaging without the need for dual-source or rapid kVp switching techniques [5].

More recently, advances in photon-counting detector CT (PCD-CT) offer a next-generation approach to functional lung imaging. Unlike conventional energy-integrating detectors, photon-counting detectors count and bin individual x-ray photons by energy, improving contrast-to-noise ratios, spatial resolution, and material decomposition while reducing beam hardening and radiation dose ^[2]^. Early investigations demonstrate that PCD-CT provides perfusion maps with image quality comparable or superior to DECT, often at lower radiation exposure [6]. As PCD-CT becomes more clinically accessible, it shows potential to unify anatomic and functional assessment in a single modality with reduced radiation dose.

Despite these advantages, there is limited clinical validation comparing PCD-CT perfusion maps directly with V/Q scans. One study by Kerber et al. studied this comparison in patients with CTEPH and found that PCD-CT perfusion maps allow for accurate CTEPH detection and are comparable to SPECT/CT at a reduced radiation dose [7]. However, broader comparative data, particularly in mixed patient populations and in direct comparison with DECT, are lacking.

In this retrospective study, we compared the diagnostic performance of PCD-CT and DECT perfusion maps against V/Q SPECT/CT as the reference standard. We also evaluated inter-observer agreement for each modality and quantified iodine concentration in normal lung parenchyma and perfusion defects to provide reference values across detector types. To our knowledge, this is the first study to directly compare both PCD-CT and DECT with V/Q scans and to offer iodine density reference ranges for each modality.

## Materials and methods

### Study design and cohort

This single-center, retrospective study was performed with institutional review board–exempt approval, and the requirement for written informed consent was waived (IRB ID Pro00148180). The institutional radiology information system was queried to identify consecutive adult patients who underwent both a clinically indicated V/Q scan and CTPA with either photon-counting–detector CT (PCD-CT) or dual-energy (energy-integrating–detector) CT (DECT) during routine clinical care.

**Fig. 1:**
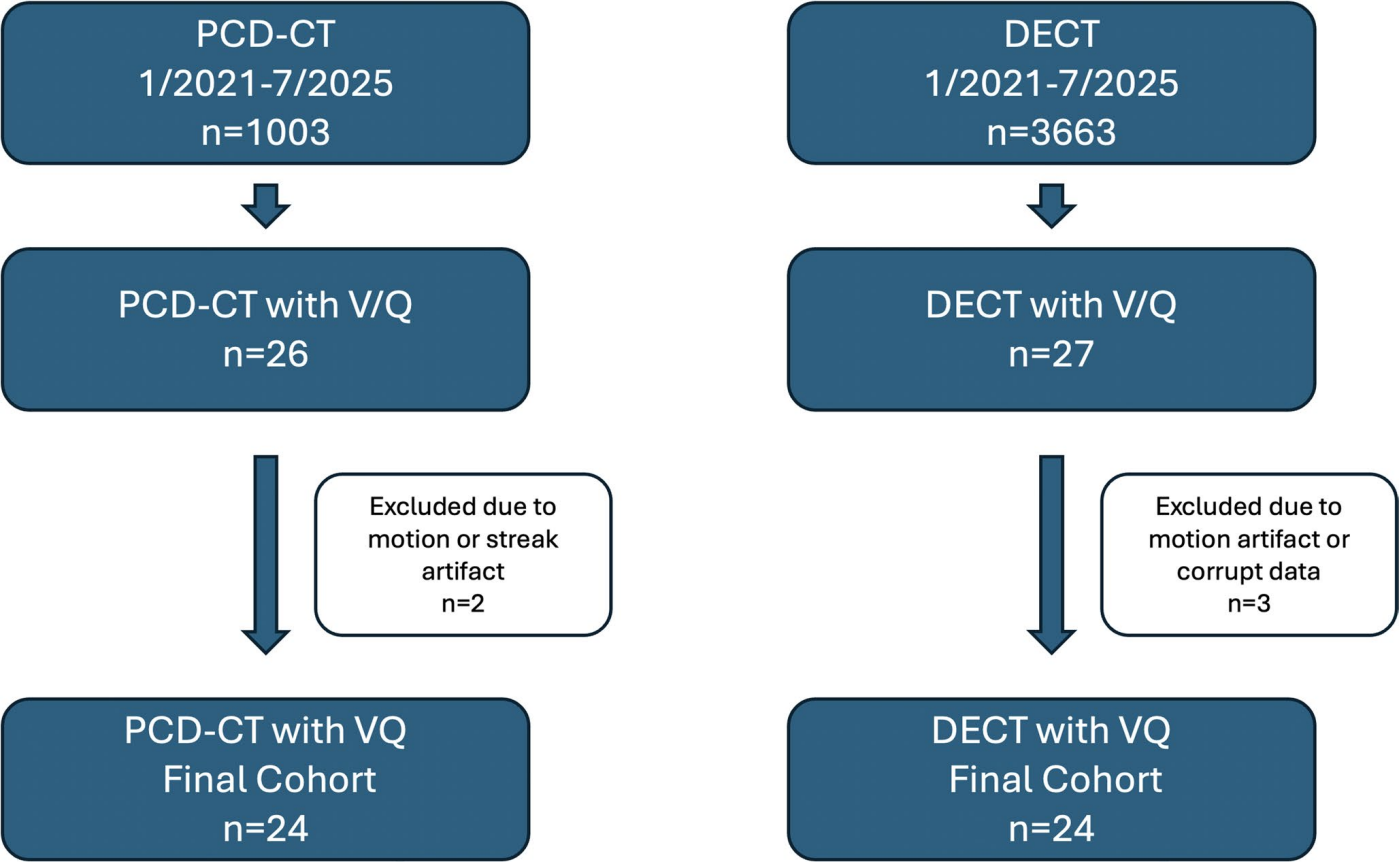
Flow diagram of patient selection. During the study period (January 2021 to July 2025), 1,526 V/Q scans, 1,003 PCD-CT examinations, and 3,663 DECT examinations were performed. For the PCD-CT cohort, 26 paired studies were identified, with 2 excluded due to nondiagnostic image quality (motion and streak artifact), resulting in 24 patients. For the DECT cohort, paired cases were identified in reverse chronological order; 3 cases were excluded (motion artifact and corrupted spectral data), resulting in 24 patients

Two cohorts were defined (Fig. 1):

- PCD-CT cohort: 24 patients who underwent V/Q scan and PCD-CT CTPA for any clinical indication.
- DECT cohort: 24 patients who underwent V/Q scan and DECT CTPA for any clinical indication.

Examinations were also excluded if CT or V/Q images were non-diagnostic because of marked motion or streak artifact. The median interval between PCD-CT and V/Q imaging was 26 days (0-367), and the median interval between DECT and V/Q imaging was 42 days (0-362). None of the patients underwent any interventional procedure or thoracic surgery, or experienced an acute clinical change (including newly diagnosed acute pulmonary embolism) between the CT and V/Q studies.

CT and V/Q scans were performed as part of routine clinical care for complementary evaluation of pulmonary vascular disease. CT provided anatomic assessment (e.g., for pulmonary embolism or pulmonary hypertension), while V/Q imaging offered functional evaluation of regional perfusion.

### CT pulmonary angiography acquisition

#### Photon-counting–detector CT

All PCD-CT examinations were performed on a first-generation dual-source photon-counting–detector CT system (e.g., NAEOTOM Alpha, Siemens Healthineers, Forchheim, Germany) using a standardized CTPA protocol. Scans were acquired in the supine position during a mid-inspiratory breath-hold in caudocranial direction.

Typical scan parameters were:

- Tube potential: 140 kVp
- Tube current: automatic tube current modulation (CARE keV IQ level of 70) with reference mAs adjusted to patient size
- Collimation: 144 × 0.4 mm with ultra-high resolution detector configuration
- Pitch: 2.0 (helical acquisition)
- Rotation time: 0.25 s
- Field of view: tailored to include entire lungs from lung apices through costophrenic angles

Raw datasets were reconstructed with a medium-sharp lung kernel suitable for perfusion analysis with:

- Slice thickness: 1 mm with 1 mm increment
- Kernel: Br40
- Iterative reconstruction: Quantum IR strength 3
- Spectral Post-Processing (SPP) data: retained for iodine/perfusion mapping and material-decomposition reconstructions

**Fig. 2:**
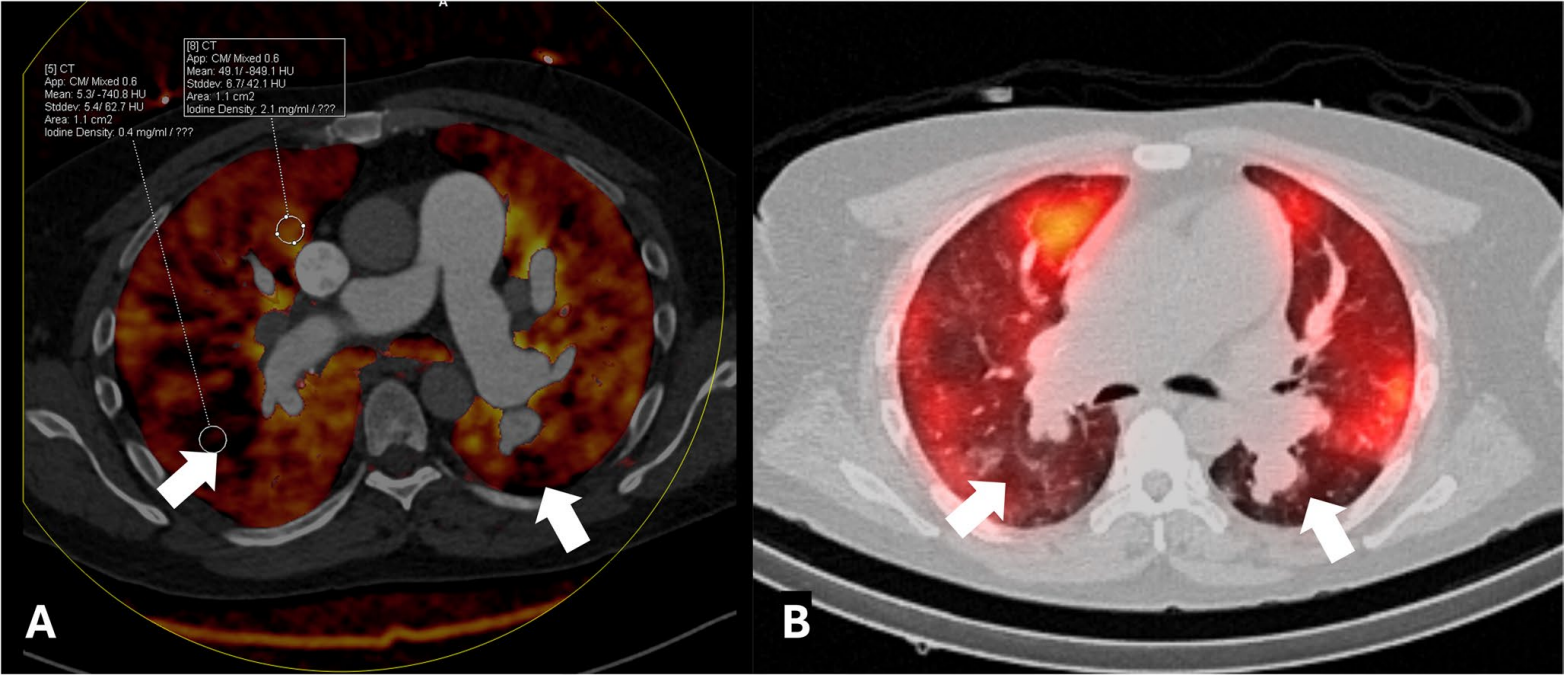
Photon-counting–detector CT iodine map vs. VQ scan. A 65-year-old man with chronic thromboembolic pulmonary hypertension. **A** Photon-counting–detector CT iodine map demonstrates multifocal lobar perfusion defects (white arrow). Quantitative iodine measurements show markedly reduced iodine density within the perfusion defect (0.2 mg/mL) compared with normally perfused lung (1.9 mg/mL). **B** Same-day V/Q SPECT perfusion imaging in the axial plane demonstrates corresponding perfusion defects in the same regions (white arrow), confirming true perfusion abnormalities

Perfusion maps were generated using Syngo.via (version VB80, Siemens Healthineers), identical to the workflow used for DECT perfusion map analysis (Fig. 2).

#### Dual-energy CT (DECT)

All DECT examinations were performed on a third-generation dual-source CT system (Somatom Force, Siemens Healthineers, Forchheim, Germany) using a standardized CTPA dual-energy protocol. Scans were acquired in the supine position during a mid-inspiratory breath-hold in caudocranial direction.

Typical scan parameters were:

- Tube potentials: 90 kVp (tube A) and 150 kVp with tin filtration (tube B)
- Tube current: automatic tube current modulation (CARE Dose4D) with reference mAs of 60 (A) and 46 (B), adjusted for patient size
- Collimation: 192 × 0.6 mm (dual-source acquisition)
- Pitch: 0.55 (helical acquisition)
- Rotation time: 0.25 s
- Field of view: tailored to include the entire lungs from apices to costophrenic angles

Raw dual-energy datasets were reconstructed using a medium lung kernel optimized for perfusion analysis with:

- Slice thickness: 1 mm with 1 mm increment
- Kernel: Br36
- Iterative reconstruction: ADMIRE, strength level 3
- Spectral Post-Processing (SPP) data: retained for iodine/perfusion mapping and material-decomposition reconstructions

**Fig. 3:**
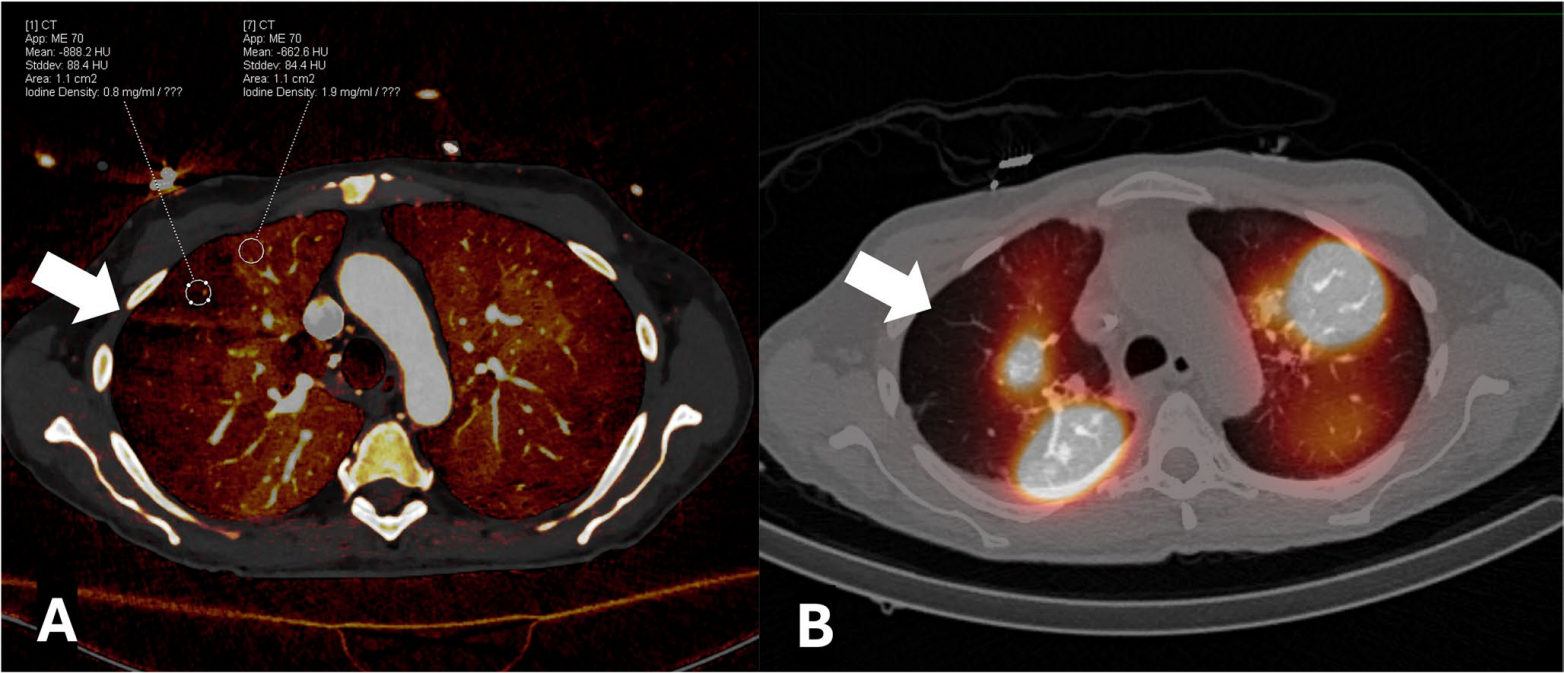
Dual energy CT perfusion blood volume map vs. VQ scan. A 61-year-old man with chronic thromboembolic pulmonary hypertension. **A** Dual energy CT perfusion blood volume map demonstrates multifocal lobar perfusion defects (white arrows). Quantitative iodine measurements show markedly reduced iodine density within the perfusion defect (0.4 mg/mL) compared with normally perfused lung (2.1 mg/mL). **B** Same-day V/Q SPECT perfusion imaging in the axial plane demonstrates corresponding perfusion defects in the same regions (white arrows), confirming true perfusion abnormalities

Perfusion maps were generated using Syngo.via (version VB80, Siemens Healthineers), identical to the workflow used for PCD-CT perfusion map analysis (Fig. 3).

#### Contrast injection and bolus timing

For both PCD-CT and DECT acquisitions, iodinated contrast medium (e.g., 350 mg I/mL) was administered intravenously using a dual-head power injector at 4.0–5.0 mL/s, followed by a 30–40 mL saline chaser at the same rate. Total contrast volume was typically 50–70 mL, adjusted for patient size. Automated bolus tracking was used with a circular region of interest (ROI) placed in the main pulmonary artery; scanning was triggered when attenuation reached 150 HU above baseline, with a fixed additional delay of approximately 4 s.

#### Ventilation–perfusion (V/Q) SPECT/CT acquisition

All V/Q examinations were performed on a dual-head gamma camera equipped with low-energy high-resolution collimators and integrated low-dose CT for SPECT/CT (Symbia T6 or Symbia Intevo Bold T16 SPECT/CT, Siemens Healthineers, Germany).

Planar ventilation images were obtained following inhalation of 555–1110 MBq (15–30 mCi) of Xenon-133. Perfusion SPECT imaging was then performed immediately following intravenous administration of around 148 MBq (4 mCi ) of Tc-99 m macroaggregated albumin.

Using the same gamma camera and patient position, a 360° SPECT acquisition was performed with:

- 60–120 projections
- 8–12 s per projection
- 128 × 128 matrix

Reconstruction of perfusion SPECT datasets was performed using ordered-subset expectation maximization with attenuation and scatter correction based on the low-dose CT component.

Low-dose CT was acquired using a non-contrast helical protocol from the lung apices through the costophrenic angles for attenuation correction and anatomic localization, during quiet tidal respiration conditions with the following parameters: collimation 6 × 1.25 mm for the Symbia T6 and 16 × 1.5 mm for the Symbia Intevo Bold T16, 110 kVp and enabled CARE Dose (mA modulation), pitch 0.75 for the Symbia T6 and 0.8 for the Symbia Intevo Bold T16, and Gantry rotation time 0.6 s for all.

### Image analysis

#### Iodine density measurements

For both PCD-CT and DECT perfusion maps, quantitative iodine density measurements were obtained at the lobar level. In each patient, circular regions of interest (ROIs) were manually placed on perfusion maps:

- Within areas of subjectively normal perfusion, avoiding segmental and subsegmental pulmonary arteries and central bronchi
- Within representative areas of perfusion defect identified on the same perfusion maps

ROIs were placed in consensus using the perfusion maps and co-registered CT images to avoid large vessels, pleura, or motion artifacts. ROIs were standardized to approximately 3 cm in diameter. In cases of heterogeneous perfusion, ROIs were positioned within the most representative areas while avoiding inclusion of mixed perfusion patterns. For each ROI, mean iodine concentration (mg/mL) and standard deviation were recorded from the workstation (Syngo.via VB80). For each patient and modality, average iodine concentration values were then calculated across normal lobes and across lobes with perfusion defects.

### Outcomes

For each modality (PCD-CT and DECT), lobar perfusion scores were compared with the consensus V/Q SPECT/CT reference standard to determine:

- Accuracy
- Sensitivity
- Specificity
- Positive predictive value (PPV)
- Negative predictive value (NPV)

Iodine density measurements were summarized separately for normally perfused lobes and lobes with perfusion defects for both PCD-CT and DECT.

### Statistical analysis

All statistical analyses were performed using R software (version 4.5.2; R Foundation for Statistical Computing, Vienna, Austria).

Lobar perfusion scores for PCD-CT and DECT were compared with the V/Q consensus as the reference standard. For each CT modality, accuracy, sensitivity, specificity, PPV, and NPV were calculated with corresponding 95% confidence intervals (CIs). Independent-sample proportion testing was used to compare PCD-CT and DECT.

For iodine density, mean values and standard deviations were calculated for normal lobes and perfusion-defect lobes for each modality. Normality of iodine density measurements was assessed using the Shapiro–Wilk test and found to be normally distributed for both PCD-CT and DECT datasets; therefore, comparisons between normally perfused regions and perfusion-defect regions were performed using paired t-test with 95% CIs reported. Iodine density values between PCD-CT and DECT (normal regions and perfusion defects) were compared using unpaired t-test to assess for systematic differences.

Inter-reader agreement for lobar perfusion scoring with PCD-CT, DECT, and V/Q scans was quantified using Cohen’s κ statistic with 95% CIs. Agreement strength was interpreted as slight (κ < 0.20), fair (0.21–0.40), moderate (0.41–0.60), substantial (0.61–0.80), or almost perfect (0.81–1.00).

A post-hoc power analysis confirmed that the sample size was sufficient to support the primary comparative analyses. Using the observed discordant accuracy counts (PCD-CT: 102/120; DECT: 107/120), the study achieved > 90% power (α = 0.05) to detect the observed differences in accuracy between modalities. Inter-reader agreement analyses also demonstrated adequate power, with > 99% power to detect a Cohen’s κ ≥ 0.70 for PCD-CT and V/Q interpretation and 86% power for DECT. Confidence intervals for sensitivity, specificity, and accuracy were within accepted precision thresholds (± 0.08–0.12), supporting the adequacy of the sample size.

Because perfusion scoring was performed at the lobar level, multiple observations were obtained from each patient. Clustering by patient was not explicitly accounted for in the statistical analysis, and therefore, confidence intervals may be slightly underestimated due to within-patient correlation.”

A two-sided p value < 0.05 was considered statistically significant for all analyses.

### Lobar perfusion scoring

All CT perfusion maps (PCD-CT and DECT) and V/Q scans were independently reviewed by two radiologists (reader 1 with 4 years’ experience; reader 2 with 14 years’ experience). Before formal data collection, 5 cases from the PCD-CT cohort and 5 cases from the DECT cohort were jointly reviewed to harmonize interpretation criteria and establish consensus examples of normal perfusion and perfusion defects.

For the main analysis, readers were blinded to each other’s scores but aware that all patients had both CT and V/Q examinations. CT perfusion maps were scored first, followed by V/Q scans. For each modality, perfusion was assessed at the lobar level. Each lobe was classified using a binary scheme:

- Positive: perfusion defect present in that lobe
- Negative: normal or homogeneous lobar perfusion without defect

Readers had access to corresponding diagnostic CTPA images for anatomic correlation when interpreting CT perfusion maps and to clinical reports while reading V/Q scans, reflecting typical clinical practice. After completion of independent scoring, both readers re-reviewed all cases in a consensus session to generate a final reference interpretation for each modality. These consensus lobar scores for V/Q SPECT/CT were used as the reference standard for calculation of diagnostic performance.

Perfusion scoring was performed at the lobar level. Each patient contributed five lung lobes (right upper, right middle, right lower, left upper, and left lower), resulting in a total of 120 lobar assessments per cohort.

## Results

### Patient demographics

The PCD-CT cohort included 24 patients (8 male, 16 female; 14 Black, 10 White) with a mean age of 49 years and mean BMI of 33.4 kg/m². Indications included CTEPH (*n* = 8), sickle cell disease (*n* = 8), evaluation for pulmonary embolism (*n* = 4), pulmonary hypertension (*n* = 3), and dyspnea with prior malignancy (*n* = 1).

The DECT cohort included 24 patients (11 male, 13 female; 11 Black, 10 White, 3 Asian) with a mean age of 56 years and mean BMI of 28.4 kg/m². Indications included CTEPH (*n* = 9), pulmonary hypertension (*n* = 9), history of lung transplantation (*n* = 2), sickle cell disease (*n* = 2), and dyspnea (*n* = 2). Cohort characteristics are summarized in Table 1.

**Table 1:** Study cohort characteristics

Parameter	PCD-CT cohort	DECT cohort
Sex		
Male	8	11
Female	16	14
Ethnicity		
White	10	10
Black	14	11
Asian	0	3
Average BMI (kg/m^2^)	33.4	28.4
Pathology		
CTEPH	8	9
Sickle Cell Disease	8	2
PE	4	0
Pulmonary Hypertension	3	9
Dyspnea	1	2
Lung transplant	0	2

The two cohorts differed in underlying clinical indications, with a higher proportion of pulmonary hypertension in the DECT cohort and greater representation of sickle cell disease and pulmonary embolism in the PCD-CT cohort. These differences reflect the independent cohort design and may influence comparative interpretation of diagnostic performance.

### Diagnostic performance compared with V/Q SPECT/CT

Using V/Q SPECT/CT as the reference standard, PCD-CT demonstrated an accuracy of 0.85 (95% CI, 0.77–0.91), with a sensitivity of 0.71 ( 95% CI, 0.56–0.82) and a specificity of 0.97 (95% CI, 0.88–0.99). The positive predictive value (PPV) was 0.95 (95% CI, 0.83–0.99) and the negative predictive value (NPV) was 0.80 (95% CI, 0.69–0.88). DECT demonstrated an accuracy of 0.89 (95% CI, 0.82–0.94), with a sensitivity of 0.52 (95% CI, 0.30–0.74) and a specificity of 0.97 (95% CI, 0.91–0.99). The PPV and NPV for DECT were 0.79 (95% CI, 0.49–0.95) and 0.91 (95% CI, 0.83–0.95), respectively. When comparing modalities directly using independent-sample proportion testing, no statistically significant differences were observed in accuracy (*p* = 0.42), sensitivity (*p* = 0.18), specificity (*p* = 0.99), PPV (*p* = 0.07), or NPV (*p* = 0.07). Although PCD-CT demonstrated numerically higher sensitivity and PPV and DECT demonstrated higher NPV, these differences did not reach statistical significance (Table 2).

**Table 2:** Diagnostic performance of PCD-CT and DECT compared with V/Q SPECT/CT

Modality	Accuracy	Sensitivity	Specificity	PPV	NPV
PCDCT	0.85 (95% CI, 0.77–0.91)	0.71 (95% CI, 0.56–0.82)	0.97 (95% CI, 0.88–0.99)	0.95 (95% CI, 0.83–0.99)	0.80 (95% CI, 0.69–0.88)
DECT	0.89 (95% CI, 0.82–0.94)	0.52 (95% CI, 0.30–0.74)	0.97 (95% CI, 0.91–0.99)	0.79 (95% CI, 0.49–0.95)	0.91 (95% CI, 0.83–0.95)
*P*-value	0.42	0.18	0.99	0.07	0.07

### Inter-reader agreement

Inter-reader agreement for lobar perfusion assessment was highest for V/Q SPECT/CT, with a Cohen’s κ of 0.87 (95% CI, 0.78–0.96), indicating almost perfect agreement. PCD-CT also demonstrated strong reproducibility, with a Cohen’s κ of 0.80 (95% CI, 0.69–0.91), reflecting substantial to almost perfect agreement between readers. In contrast, DECT showed moderate inter-reader agreement, with a Cohen’s κ of 0.59 (95% CI, 0.38–0.80). Overall, these findings indicate that PCD-CT provides more consistent interpretation across readers compared with DECT, and that agreement with V/Q SPECT/CT remains highest among the three modalities. Inter-reader agreement results are summarized in Table 3.

**Table 3:** Inter-reader agreement for lobar perfusion assessment

	PCD-CT	DECT	V/Q
Cohen’s kappa	0.80	0.59	0.87

### Iodine density measurements

For PCD-CT, iodine density was significantly higher in normally perfused lobes (mean 2.1 ± 0.29 mg/mL) compared with perfusion-defect lobes (0.40 ± 0.18 mg/mL; *p* < 0.001). For DECT, iodine density was similarly higher in normal lobes (2.3 ± 0.29 mg/mL) compared with perfusion-defect lobes (0.49 ± 0.17 mg/mL; *p* < 0.001).

Between-modality comparisons showed no significant difference in iodine density for normally perfused lung (*p* = 0.94) or for perfusion-defect regions (*p* = 0.68), indicating consistency across both platforms (Table 4).

**Table 4:** Quantitative iodine concentration in normal and hypoperfused lung

Modality	Normal perfusion (mg/mL)	Perfusion defect (mg/mL)	*P*-value
PCDCT	2.10 ± 0.29	0.40 ± 0.18	2.2 × 10⁻¹¹
DECT	2.30 ± 0.29	0.49 ± 0.17	3.3 × 10⁻⁹

## Discussion

This study evaluated the diagnostic performance of PCD-CT and DECT perfusion mapping using V/Q SPECT/CT as the reference standard. Across two independent cohorts, both PCD-CT and DECT demonstrated high overall accuracy and specificity for detecting lobar perfusion defects, with no statistically significant differences between modalities. Sensitivity was numerically higher for PCD-CT, whereas DECT yielded slightly higher negative predictive value, although neither difference reached statistical significance. Inter-reader agreement was substantial to almost perfect for PCD-CT and V/Q SPECT/CT, and moderate for DECT. Additionally, iodine density values in normally perfused lung and perfusion-defect regions were highly distinct for both PCD-CT and DECT, and no significant differences in iodine concentration were observed between modalities.

The diagnostic performance of PCD-CT in our study is consistent with prior work demonstrating that spectral CT techniques can reliably detect regional perfusion abnormalities. In our cohort, PCD-CT achieved an accuracy of 85%, with a sensitivity of 71%, specificity of 97%, and substantial inter-reader agreement (κ = 0.80). Only one prior study has directly compared PCD-CT perfusion maps with V/Q scan: Kerber et al. reported that PCD-CT iodine maps demonstrated high sensitivity (94%), specificity (84%), and overall accuracy (88%) for the diagnosis of chronic pulmonary thromboembolism, with good inter-reader agreement (κ = 0.74). Their study focused primarily on patients with chronic thromboembolic pulmonary hypertension, a population with larger, well-defined segmental or lobar perfusion defects that are more easily detected on CT iodine maps. In contrast, our cohort included a broader mix of indications, including pulmonary hypertension of mixed etiology, sickle cell disease, and dyspnea, which introduces more subtle or heterogeneous perfusion patterns that may reduce sensitivity. Despite these differences, the overall diagnostic accuracy and high specificity observed in our study reinforce the robustness of PCD-CT perfusion mapping across diverse clinical settings.

The diagnostic performance of DECT perfusion mapping in our study is also broadly consistent with previously published work. DECT demonstrated an overall accuracy of 89%, with a sensitivity of 52%, specificity of 97%, and moderate inter-reader agreement (κ = 0.59). Thieme et al. reported per-segment sensitivity and specificity of 83% and 99%, respectively, with strong negative predictive value, while noting peripheral coverage limitations with early-generation DECT hardware [8]. In patients with acute pulmonary embolism, iodine map CTPA demonstrated a segment-based sensitivity of 81% and specificity of 79% when compared with perfusion SPECT, with the majority of false-positive and false-negative findings attributable to well-recognized artifacts [9]. In CTEPH-specific cohorts, DECT has performed particularly well, with studies reporting patient-level sensitivity of 100% and specificity of 92–93% for identifying perfusion defects relative to V/Q scanning [10]. The lower sensitivity in our cohort likely reflects the broader spectrum of clinical indications, including nonthromboembolic pulmonary hypertension, sickle-related microvascular abnormalities, and mixed parenchymal disease. Nonetheless, the consistently high specificity across studies and in our data reinforces DECT’s value as a reliable tool for confirming perfusion defects within a standard CTPA examination.

In our study, PCD-CT and DECT demonstrated comparable overall diagnostic accuracy for detecting lobar perfusion defects, with no statistically significant differences between modalities. PCD-CT showed numerically higher sensitivity, which may reflect the intrinsic advantages of photon-counting detector technology, including smaller detector pixel size and improved spatial resolution that enhance the delineation of subtle, heterogeneous perfusion abnormalities [11, 12]. Conversely, DECT exhibited a slightly higher negative predictive value, a finding that may relate to its acquisition of two distinct energy spectra, which provides robust material decomposition and may improve uniformity of iodine signal in normally perfused lung [13]. Although these differences did not reach statistical significance, they highlight complementary strengths of the two techniques. Notably, PCD-CT generates spectral perfusion maps without additional radiation exposure, representing a meaningful advantage over DECT, which requires dual-energy acquisition. Furthermore, PCD-CT technologies continue to evolve, and future improvements in energy binning and spectral reconstruction may further enhance both sensitivity and quantitative fidelity of PCD-based perfusion assessment.

The findings support the role of spectral CT perfusion as a reliable tool for functional pulmonary assessment that can be integrated into routine CTPA without additional acquisition. From both emergency radiology and pulmonary medicine perspectives, this capability has important clinical implications. In acute care settings, where rapid diagnosis is essential and V/Q imaging may be limited by availability or time constraints, spectral CT–based perfusion imaging may reduce reliance on additional functional studies by providing both anatomic and functional information within a single examination. This approach may be particularly valuable in patients with distal or small-vessel pulmonary vascular disease, such as CTEPH and sickle cell disease, where conventional CTPA may be limited in detecting subtle or peripheral perfusion abnormalities. In these scenarios, the addition of iodine-based perfusion maps can enhance detection of functional deficits, improve diagnostic confidence, and provide a more comprehensive assessment of disease burden. Prior studies have similarly demonstrated that CT perfusion imaging can enhance diagnostic precision in complex pulmonary conditions, including acute chest syndrome in sickle cell disease [4].

Importantly, perfusion mapping is not intended to replace conventional CTPA interpretation based on visualization of intravascular filling defects. Rather, it serves as a complementary functional tool that augments anatomic assessment and may improve diagnostic accuracy in selected patient populations. As spectral CT technology continues to evolve and become more widely available, its integration into both emergency and pulmonary imaging workflows has the potential to streamline diagnostic pathways and reduce the need for multimodality imaging in appropriately selected cases.

Inter-reader agreement in our study demonstrated important differences between modalities and provides additional insight into clinical interpretability. PCD-CT showed substantial to almost perfect agreement (κ = 0.80), closely approaching the reproducibility of V/Q SPECT/CT (κ = 0.87). This high level of agreement is consistent with the only prior PCD-CT perfusion study, where Kerber et al. reported good inter-reader agreement (κ = 0.74), supporting the notion that photon-counting detector technology produces perfusion maps with reliable interpretive consistency [7]. For DECT, our study demonstrated moderate agreement (κ = 0.59), a finding that closely mirrors results from prior dual-energy CT literature. In a prospective by Chae J et al. assessing DECT perfusion defect scoring in acute pulmonary embolism, the readers achieved moderate to substantial agreement (segmental κ values of 0.51–0.56), nearly identical to the agreement levels observed in our cohort [14]. These converging results suggest that DECT iodine maps, while diagnostically valuable, remain more prone to variability. In contrast, the higher agreement observed with PCD-CT may reflect its improved signal to noise ratio, contrast to noise ratio and spatial resolution which collectively minimize interpretive ambiguity [12]. Given that perfusion mapping often informs subsequent diagnostic and therapeutic decisions, the greater reproducibility seen with PCD-CT represents a clinically meaningful advantage.

Quantitative analysis of iodine density provided additional insight into the behavior of spectral perfusion maps across detector platforms. In both PCD-CT and DECT, iodine concentration was substantially higher in normally perfused lung compared with lobar perfusion defects, with clear separation between groups and highly significant differences. These trends are consistent with the underlying physiology of pulmonary perfusion and support the biological validity of iodine-based spectral maps. Importantly, iodine density values for normal and hypoperfused lung were similar between PCD-CT and DECT, suggesting that despite differences in detector technology and spectral acquisition, both platforms yield comparable quantitative perfusion metrics at the lobar level.

An important consideration in interpreting our findings is that the PCD-CT and DECT groups consisted of independent cohorts with differing clinical compositions. The DECT cohort included a higher proportion of patients with pulmonary hypertension, whereas the PCD-CT cohort included more patients with sickle cell disease and pulmonary embolism. These conditions are associated with distinct patterns of perfusion abnormality, ranging from more diffuse or heterogeneous changes to more focal segmental or lobar defects. As a result, differences in disease distribution may influence both the detectability of perfusion abnormalities and inter-reader agreement. Accordingly, the observed differences in diagnostic performance between modalities should be interpreted with caution.

To our knowledge, no prior study has reported quantitative iodine density values for pulmonary perfusion assessment using photon-counting CT, making our findings the first to define reference measurements for both normal and hypoperfused lung parenchyma on this platform. In our PCD-CT cohort, normally perfused lobes demonstrated a mean iodine density of 2.10 ± 0.29 mg/mL, compared with 0.40 ± 0.18 mg/mL in perfusion-defect regions. Similarly, DECT showed 2.30 ± 0.29 mg/mL in normal lung versus 0.49 ± 0.17 mg/mL in defective areas, values that closely align with prior DECT literature reporting normal iodine concentrations of approximately 1.5 mg/mL in healthy lung [15]. Such measurements are known to vary across institutions due to differences in contrast protocols, patient factors, and spectral reconstruction techniques, further supporting the importance of providing detailed acquisition parameters and reporting modality-specific reference ranges. By establishing quantitative iodine values for both normal and abnormal perfusion across two spectral CT platforms, our study offers practical benchmarks that may aid reader training, support threshold development for automated or AI-assisted analysis, and promote greater standardization of quantitative spectral perfusion imaging.

This study has several limitations. First, its retrospective, single-center design and relatively small sample size may limit generalizability, particularly given the heterogeneous clinical indications represented in each cohort. Second, although both PCD-CT and DECT groups were compared with V/Q SPECT/CT, the patient cohorts were independent, preventing direct, within-patient comparison of the two CT techniques. Third, our analysis was performed at the lobar rather than segmental level. Fourth, image quality and iodine quantification can be influenced by institutional contrast protocols, spectral reconstruction methods, and vendor-specific post-processing, which may differ from other centers. Finally, although our study provides the first quantitative iodine density reference values for PCD-CT perfusion imaging, these values may evolve as photon-counting technology advances and as additional energy bins and reconstruction algorithms become available. Future studies with larger, multi-institutional cohorts and standardized acquisition parameters will be important to further validate these findings.

## Conclusion

In conclusion, both photon-counting CT and dual-energy CT demonstrated high diagnostic accuracy and specificity for identifying lobar perfusion defects when compared with V/Q SPECT/CT. PCD-CT showed slightly higher sensitivity and higher inter-reader agreement, while DECT provided marginally higher negative predictive value, although these differences were not statistically significant and the results should be interpreted in the context of the independent cohort design. Quantitative iodine measurements showed clear separation between normal and hypoperfused lung for both modalities, and our study provides the first reference values for PCD-CT perfusion analysis.

These findings highlight the potential of spectral CT techniques to serve as robust functional complements to conventional CT pulmonary angiography by providing both anatomic and perfusion information within a single examination. This capability may be particularly valuable in the evaluation of distal or small-vessel pulmonary vascular disease, such as chronic thromboembolic pulmonary hypertension and sickle cell disease, where conventional CT may be limited in detecting subtle perfusion abnormalities. Beyond pulmonary vascular disease, spectral CT–based perfusion imaging may also have broader applications, including assessment of tumor perfusion, treatment response, and other conditions involving regional pulmonary blood flow abnormalities. As spectral imaging continues to evolve, further studies are needed to define its role across these clinical domains and to establish standardized approaches for quantitative perfusion assessment.

## Data Availability

Data generated or analyzed in this study are available from the corresponding author upon reasonable request.

## Abbreviations

CI: Confidence interval
CT: Computed tomography
CTPA: CT pulmonary angiography
CTEPH: Chronic thromboembolic pulmonary hypertension
DECT: Dual—energy CT
mg/mL: Milligrams per milliliter
NPV: Negative predictive value
PCD: CT—Photon—counting—detector CT
PE: Pulmonary embolism
PPV: Positive predictive value
SD: Standard deviation
SPECT: Single—photon emission computed tomography
V/Q SPECT/CT: Ventilation—perfusion SPECT with CT attenuation correction
